# Dysphagia Lusoria Causing Aspiration Pneumonitis in a Patient With Recurrent Pancreatitis

**DOI:** 10.7759/cureus.30635

**Published:** 2022-10-24

**Authors:** Bharadwaj Adithya Sateesh, Nicole Gousy, Yogesh Prajapati, Girma M Ayele, Miriam B Michael

**Affiliations:** 1 Medicine, University of Maryland Midtown Campus, Baltimore, USA; 2 Medicine, American University of Antigua, St. John's, ATG; 3 Medicine, American University of Antigua, New York City, USA; 4 Internal Medicine, American University of Antigua, St. John's, ATG; 5 Internal Medicine, Howard University Hospital, Washington DC, USA; 6 Internal Medicine, University of Maryland Midtown Campus, Baltimore, USA

**Keywords:** dysphagia lusoria, arsa esophageal compression, recurrent aspiration, arsa, aberrant rigt subclavian artery

## Abstract

Dysphagia lusoria (DL) occurs due to an aberrant right subclavian artery (ARSA) compressing the esophagus resulting in dysphagia, odynophagia, and/or reflux symptoms. It is diagnosed by barium esophagram followed by a CT scan or MRI. In this case report, there is a 44-year-old male with a chronic history of reflux and a cough that presents after a meal. The case was complicated due to the history of the patient's alcoholism with recurrent pancreatitis. A CT scan was obtained during his admission, which showed pleural effusion, atelectasis, aspiration pneumonia, and an incidental aberrant RCA. Although DL is a rare pathology, 60%-80% of patients remain asymptomatic, and patients with symptoms can be managed conservatively or surgically, depending on their severity.

## Introduction

​​Dysphagia lusoria (DL), otherwise known as Bayford-Autenrieth dysphagia, is a rare embryological abnormality that occurs during the development of the aortic arch and its branches [1]. During normal embryological development, there are effectively two aortic arches, with the right subclavian artery branching off from the right fourth aortic arch and the seventh cervical intersegmental artery. However, the right aortic arch eventually becomes atretic and forms into the right innominate artery [2]. Rarely an aberrant right subclavian artery (ARSA) can result from a failure of involution of the fourth vascular arch after the origin of the left subclavian artery [3]. This formation typically projects posterior to the esophagus. However, there are rare cases of an ARSA anteriorly coursing between the esophagus and trachea or simply coursing anterior to the trachea [3,4]. Approximately 80% of ARSAs project between the esophagus and the vertebral column, 15% of ARSAs course between the esophagus and the trachea, and only 5% of ARSAs run anteriorly to the trachea and the esophagus [2]. Commonly, an ARSA is complicated by having a wide base, known as Kommerell’s diverticulum, which can widen in the context of aging [5]. The diagnosis of dysphagia lusoria is characterized as having esophageal compression, with resulting dysphagia, odynophagia, and/or reflux secondary to an ARSA, such as in the patient reported herein. It is estimated that in a general population, the prevalence of DL is 0.4%-0.7%, and the incidence of ARSA is 0.5%-1.8% [2,6]. Since 60%-80% of patients with an ARSA will remain asymptomatic throughout their lifetime, the diagnosis of DL is incredibly rare [2,7]. ARSA’s can be found incidentally during esophageal tests (barium esophagram) while evaluating for other causes [2,7,8]. Once an ARSA is confirmed with a CT scan or MRI, patients are managed either conservatively or with surgical vascular decompression [2,7]. We present a case of DL diagnosed from an incidental ARSA found on admission.

## Case presentation

A 44-year-old male with a past medical history of recurrent episodes of pancreatitis, aspiration pneumonia, and alcohol use disorder presented to our emergency department due to worsening chronic abdominal pain exacerbated after alcohol intake. He also complained of a long-standing history of reflux, for which he is on omeprazole 40mg but has no relief. On further questioning, he mentioned a cough that always manifests after eating. He also reported a history of alcoholic pancreatitis. 

**Figure 1:**
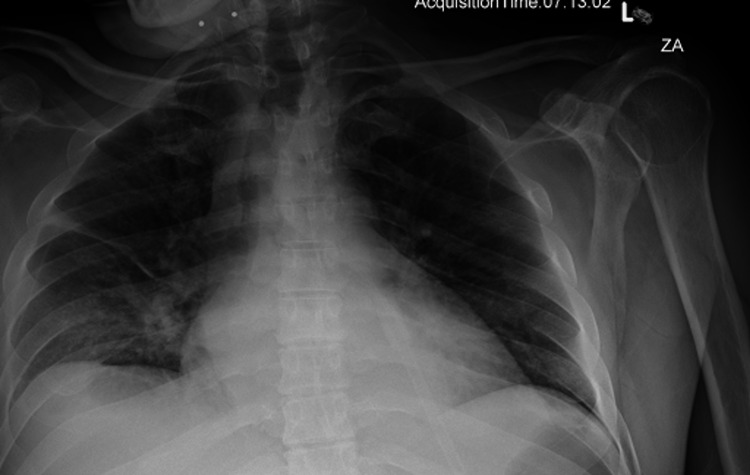
Chest X-ray Chest X-ray showing a right mid-lower lung zone opacities could represent infection and/or aspiration.

On investigation, the patient's lipase was found to be 722 U/L. Urine toxicology was positive for opiates, and ethanol was 334 mg/dL. He was admitted with the diagnosis of pancreatitis and was managed symptomatically with IV fluid. The patient remained stable till the third day of admission but started desaturating overnight with oxygen saturation in the mid-80s, and an X-ray showed right lung field opacity (Figure 1). With the diagnosis of aspiration pneumonitis, he was put on nasal oxygen. During this assessment, the patient was alert and oriented x 3. Reported mild intermittent sharp chest pain with a pain score of eight out of 10; the pain is reproducible. Also complained of epigastric pain, nausea, and one episode of vomiting and back pain. 

**Figure 2:**
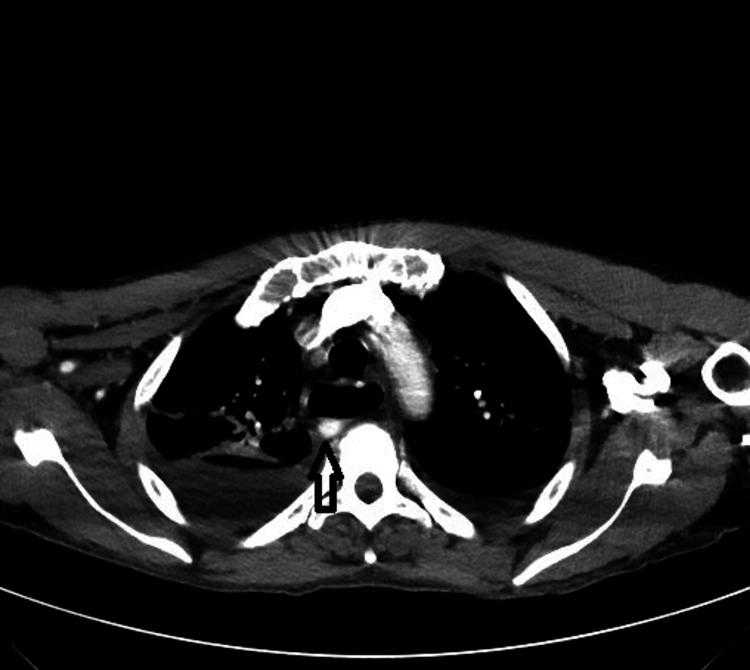
Chest CT angiography Chest CT angiography showing aberrant right subclavian artery (arrow)

**Figure 3:**
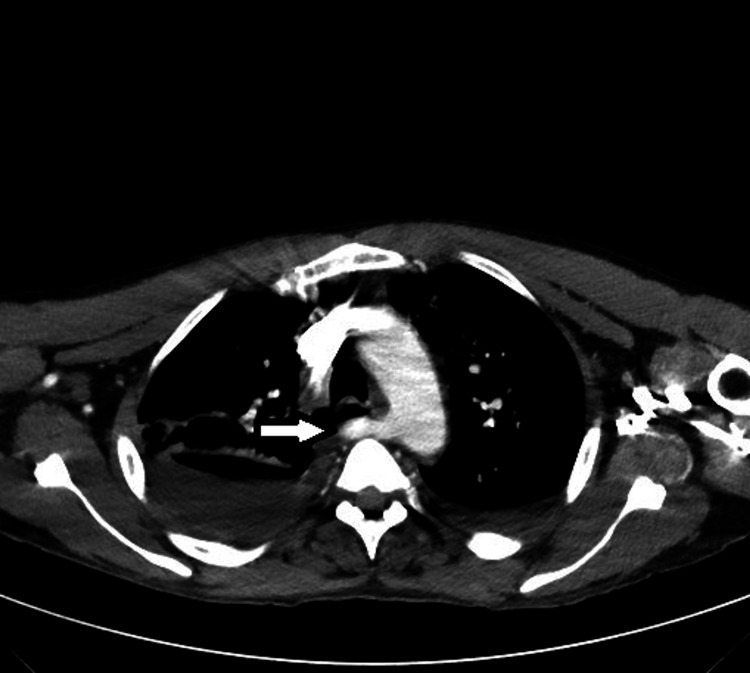
Chest CT angiography Chest CT angiography showing an aberrant right subclavian artery (arrow) arising from the right subclavian artery

**Figure 4:**
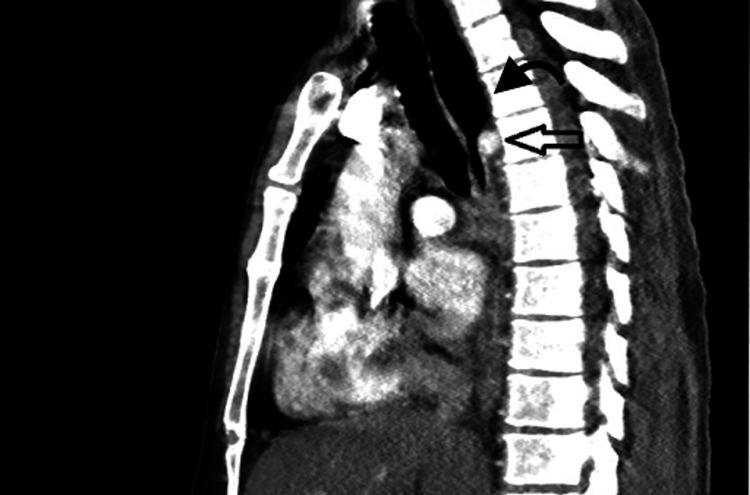
Chest CT angiography Chest CT angiography shows aberrant right subclavian artery (arrow), causing a mild mass effect on the upper esophagus and dilated patulous esophagus (curved black arrow) containing fluid with diffuse circumferential wall thickening in the mid and distal aspects.

The patient continued to be hypoxic and required oxygen via nasal cannula. The patient underwent a CT chest which showed moderate bilateral pleural effusions, some lower lobe atelectasis, and possibly some debris in the airways. Also incidentally noted was an aberrant right subclavian artery causing some compression of his esophagus with marked dilation of the esophagus superior to this compression (Figures 2-4). The patient was given diuretics for his effusion which was deemed secondary to his pancreatitis/fluid resuscitation. He was referred to vascular surgery to fix his lesion, which would give him relief from regurgitation and aspiration complications. The patient declined the surgical options offered to him.

## Discussion

Dysphagia lusoria (DL) refers to progressive dysphagia that occurs secondary to esophageal and/or tracheal compression due to an ARSA [8]. While 3% of autopsy studies have shown anomalies of the aortic arch, this specific anatomical abnormality is one of the most common congenital abnormalities, with an incidence of 0.5%-1.8% within the general population [2]. There is a 37% higher prevalence of ARSA in children with other congenital cardiac diseases, such as tetralogy of Fallot (2%), pulmonary atresia (2%), or children with Trisomy 21 [2]. Although symptoms of DL tend to be rare, they present in a bimodal distribution during early childhood and within the fifth decade of life. Both distributions have a female predominance [8]. It is thought that late-onset symptoms of DL are attributed to progressive esophageal stiffness and decreased motility as a product of aging. Additionally, age-related atherosclerosis can lead to vascular alterations, including ARSA stiffening, aortic elongation, or dilation of Kommerell’s diverticulum, all of which can contribute to more pronounced esophageal compression [1,5].

Interestingly, however, 60%-80% of patients with ARSA remain asymptomatic within their lifetime, with an ARSA being an incidental finding on autopsy, making the diagnosis of DL exceedingly rare as only 20%-40% of those with an ARSA develop DL [7]. While there have been reports documenting multiple vascular structures within the chest cavity as a cause of esophageal compression, compression symptoms due to an ARSA is pathognomonic for DL [2]. The most commonly reported symptom in adults includes those associated with mechanical esophageal obstruction, progressively worsening dysphagia (91%), regurgitation of poorly chewed food, chest pain, postprandial bloating, and coughing, or Horner’s syndrome. Chest pain was one of the least reported symptoms (<20%), followed by a rare case of ruptured aneurysmal rupture of the Kommerell diverticulum [5,7]. 

In the patient reported here, his primary complaint was chronic, refractory reflux with a cough after eating. While this may be one of the earlier signs of dysphagia, it is unusual for reflux to be the initial symptom of DL rather than dysphagia. However, reflux refractory to proton pump inhibitors (PPI) should be a warning sign to physicians indicating an underlying pathology to the reflux. Thus, it is crucial for patients with chronic reflux and those who have failed PPI therapy for 8-12 months to undergo esophageal imaging and manometry along with head and neck imaging to look for physical obstructions or other causes of dysmotility [8]. 

Regardless, this patient presented with signs of aspiration pneumonia requiring supplemental oxygen. This episode of aspiration pneumonia may have dual casualties. Considering this patient’s history of chronic alcohol abuse, as highlighted by his previous history of frequent bouts of acute and chronic pancreatitis, his risk for aspiration pneumonia would generally be increased. However, his new diagnosis of DL secondary to an ARSA brings to light a second pathology that might have contributed, at least partially, to this episode of aspiration pneumonia and may contribute to future potential complications. Therefore, early DL diagnosis is crucial for managing and preventing complications regarding esophageal compressions, such as aspiration pneumonia. 

Since symptom severity widely ranges in those with DL, treatment must also vary accordingly. Mild to moderate symptoms are treated conservatively with lifestyle modifications, including smaller meals, eating slower, chewing thoroughly, and increased fluid intake with meals [2,9]. Since many patients also complain of reflux, proton pump inhibitors in combination with promotility agents were shown by Janssen et al. to improve symptoms [10]. If conservative measures are not effective, surgical treatment has also been proposed. In a literature review by Levitt, 14 out of 24 patients failed conservative management of DL and required surgery with the goal of removing the ARSA [2]. Since this surgery involves reconstruction and anastomosis of the aberrant vessel, those not considered good candidates for this surgery may require repeated endoscopic esophageal dilations over their lifetime [7]. However, due to the rarity of DL, there is still uncertainty surrounding the most effective treatment algorithm [7].

## Conclusions

Dysphagia lusoria is caused by an embryonic stage developmental anomaly of the aortic arch resulting in an ARSA, among other complications. The ARSA usually juts in between the esophagus and the vertebral column. This can lead to compression of the esophagus, leading to symptoms of dysphagia, regurgitation of food, aspiration, and reflux that is not responsive to traditional treatment options. The presence of other possible causes of aspiration and reflux, like alcoholism, complicates the approach to managing patients with long-term unresponsive reflux. We presented a case of a patient with recurrent pancreatitis due to alcohol use disorder who also had a history of reflux that was nonresponsive to conventional treatments. The patient was then incidentally found to have an ARSA, leading to the diagnosis of DL. We recommend that in patients with long-standing reflux with other possible etiologies, consideration and testing towards anatomic etiologies should be performed as well.
